# Ongoing Challenges in the Diagnosis of Myelin Oligodendrocyte Glycoprotein Antibody–Associated Disease

**DOI:** 10.1001/jamaneurol.2023.3956

**Published:** 2023-10-10

**Authors:** Patrick Lipps, Ana Beatriz Ayroza Galvão Ribeiro Gomes, Laila Kulsvehagen, Matthias Anthony Mutke, Jens Kuhle, Athina Papadopoulou, Anne-Katrin Pröbstel

**Affiliations:** 1 Department of Neurology, University Hospital of Basel and University of Basel, Basel, Switzerland; 2 Departments of Biomedicine and Clinical Research, University Hospital of Basel and University of Basel, Basel, Switzerland; 3 Research Center for Clinical Neuroimmunology and Neuroscience Basel (RC2NB), University Hospital of Basel and University of Basel, Basel, Switzerland; 4 Clinic of Radiology and Nuclear Medicine, Division of Diagnostic and Interventional Neuroradiology, University Hospital of Basel and University of Basel, Basel, Switzerland; 5 Translational Imaging in Neurology (ThINk) Basel, Department of Biomedical Engineering, University Hospital of Basel and University of Basel, Basel, Switzerland

## Abstract

This cross-sectional study examines whether proposed myelin oligodendrocyte glycoprotein antibody–associated disease (MOGAD) diagnostic criteria can exclude other diseases, such as multiple sclerosis, and rely on results of cell-based assays.

With recognition of myelin oligodendrocyte glycoprotein (MOG) antibody–associated disease (MOGAD) as a distinct entity and wider availability of MOG-IgG testing, clinicians are frequently confronted with the challenge of diagnosing MOGAD.^1^ To facilitate diagnosis, consensus-elaborated diagnostic criteria relying on the presence of MOG-IgG, core clinical events, and exclusion of differential diagnoses were proposed.^2^ The criteria require clinical interpretation to exclude other diseases, such as multiple sclerosis (MS) (challenge 1), and rely on heterogeneous assay results (challenge 2); thus, diagnoses may not be unequivocal. We investigated the reliability of the MOGAD diagnostic criteria to address these 2 challenges.

## Methods

We analyzed data from a multicenter study of patients with suspected or confirmed demyelinating diseases.^3^ Institutional review boards of participating centers approved this cross-sectional study. All patients provided written informed consent. We followed the STROBE reporting guideline.

For challenge 1, patients fulfilling diagnostic criteria for MS^4^ with positive MOG-IgG results in a live cell-based assay (CBA) were included and independently rated by 2 neurologists (A.B.A.G.R.G., A.P.) for final diagnosis (MOGAD^2^ or MS^4^). For challenge 2, patients with at least 1 core clinical demyelinating event suggestive of MOGAD^2^ and 2 independent MOG-IgG results in a live CBA and a fixed CBA were included (eMethods in Supplement 1).

Interrater reliability (Cohen κ coefficient), interassay, and intercenter diagnosic concordance rates were calculated (eMethods in Supplement 1). Data analysis was performed using Microsoft Excel 16.77 (Microsoft Corp) and RStudio 2022.07.2 with R 4.2.0 (RStudio) from January to August 2023.

## Results

We included 162 patients (121 females [74.7%], 41 males [25.3%]; mean [SD] age, 43.1 [15.3] years). Challenge 1 group included 28 patients with positive MOG-IgG results, of whom 21 (75%; 12 with clear-positive and 9 with low-positive results) received MS diagnosis, 2 (7%; 1 with clear-positive and 1 with low-positive results) MOGAD diagnosis, and 5 (18%; 2 with clear-positive and 3 with low-positive results) discordant diagnoses. Twenty-seven patients (96%) presented records of core demyelinating events that were compatible with MOGAD. All 28 patients fulfilled imaging criteria for the MS diagnosis, 21 of whom (75%) also presented at least 1 supporting magnetic resonance imaging (MRI) feature compatible with MOGAD diagnosis. All 5 patients with discordant diagnoses presented positive cerebrospinal fluid (CSF)–specific oligoclonal bands, which were usually associated with myelitis (4 [80%]), and supportive imaging features for MOGAD (Table). The interrater reliability was fair (Cohen κ = 0.35).

**Table.** Clinical Characteristics of Patients With Discordant Final Diagnoses

Patient sex	Age at disease onset	Age at sampling	Attacks, No.	KFSS, functions (score)	EDSS score	CSF-specific OCBs test result	MOG-IgG test result	Core clinical syndromes	Supporting MRI features
Male	Mid-teens	Late 30s	2	Pyramidal (3), cerebellar (3), sensory (3), brainstem (1), bowel and bladder (4), visual (1), mental (0), other (0), ambulation (6)	6.0	Positive	Clear positive	Optic neuritis and myelitis	Partially ill-defined T2 hyperintense lesions in supratentorial white matter
Female	Early 30s	Early 30s	2	Pyramidal (2), cerebellar (1), sensory (2), brainstem (1), bowel and bladder (0), visual (0), mental (2), other (0), ambulation (0)	3.0	Positive	Low positive	Cerebral monofocal or polyfocal deficits	Partially ill-defined T2 hyperintense lesions in supratentorial white matter
Female	Mid-40s	Mid-40s	1	Pyramidal (2), cerebellar (2), sensory (3), brainstem (2), bowel and bladder (1), visual (2), mental (2), other (0), ambulation (1)	4.0	Positive	Low positive	Myelitis and brainstem	LEM, central cord lesion without H sign
Female	Mid-20s	Late 40s	3	Pyramidal (3), cerebellar (2), sensory (3), brainstem (1), bowel and bladder (3), visual (0), mental (2), other (0), ambulation (1)	4.5	Positive	Clear positive	Myelitis and brainstem	LEM, conus lesion, central cord lesion without H-sign, ill-defined T2-hyperintense lesions in supratentorial white matter and cerebellar peduncles
Male	Early 50s	Early 50s	3	Pyramidal (3), cerebellar (2), sensory (2), brainstem (2), bowel and bladder (1), visual (0), mental (1), other (0), ambulation (6)	6.0	Positive	Low positive	Optic neuritis, myelitis, brainstem	LEM, central cord lesion with H sign, deep gray matter involvement

Abbreviations: CSF, cerebrospinal fluid; EDSS, Expanded Disability Status Scale; KFSS, Kurtzke Functional Systems Score; LEM, longitudinally extensive myelitis; MOG, myelin oligodendrocyte glycoprotein; MRI, magnetic resonance imaging; OCBs, oligoclonal bands.

Challenge 2 group included 134 patients, of whom 108 (81%; 97 negative, 2 low-positive, 9 clear-positive) exhibited concordant MOG-IgG results. Among 26 patients with divergent results, 17 (65%) had low-positive results in either CBA. A 90% (121 of 134) diagnostic agreement rate was observed when applying the proposed criteria to each center’s MOG-IgG results (Figure).

**Figure.**
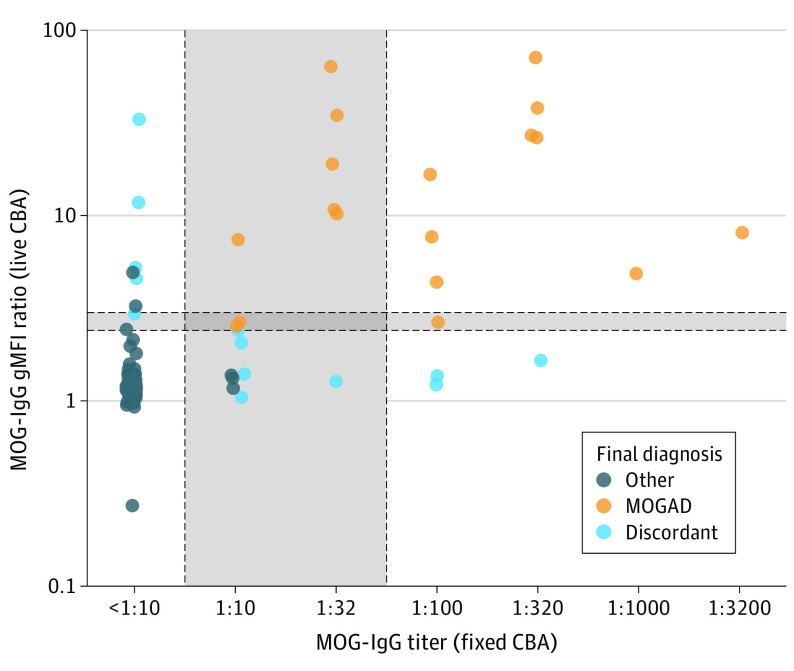
Myelin Oligodendrocyte Glycoprotein (MOG)–IgG Results With 2 Independent Cell-Based Assays (CBAs) Gray areas with dotted lines indicate low-positive range of the fixed CBA (≥1:10 to <1:100) and live CBA (≥2.4 to <3.0); orange, final diagnosis of myelin oligodendrocyte glycoprotein antibody-associated disease (MOGAD); dark blue, final diagnosis distinct from MOGAD; and light blue, discordant final diagnosis.

## Discussion

Findings revealed that the proposed diagnostic criteria in some cases may not accurately distinguish MOGAD from MS, contributing to diagnostic inconsistencies across centers. MOG-IgG testing and consideration of MOGAD diagnosis are recommended only in the absence of better alternative causes, such as MS. While this recommendation prevents unwarranted MOG-IgG testing, with potential false-positive results, the precondition cannot always be met because patients often present with overlapping clinical and imaging features. Consideration of additional laboratory, advanced MRI, or optical coherence tomography features may be needed in establishing accurate diagnoses.

Inconsistent replicability of MOG-IgG detection across assays and decreased reproducibility of low-positive results pose additional challenges. Although the panel aimed to increase comparability by categorizing antibody results as clear-positive or low-positive,^2^ additional strategies are necessary to mitigate the risk of false-positive or false-negative results. Standardized testing remains unavailable worldwide, but repeated testing of sera with alternative assays or CSF testing in cases of seronegative results may increase diagnostic certainty in patients with high disease suspicion. A study limitation was its retrospective and cross-sectional design.

The proposed criteria mark a breakthrough toward improved MOGAD diagnosis. However, additional research and validation are needed to establish standardized assays and develop novel biomarkers to refine these criteria.

## Notes

Supplement 1.
eMethods

Supplement 2.
Data Sharing Statement

## References

[1] Marignier R, Hacohen Y, Cobo-Calvo A, . Myelin-oligodendrocyte glycoprotein antibody-associated disease. Lancet Neurol. 2021;20(9):762-772. doi:10.1016/S1474-4422(21)00218-0 34418402

[2] Banwell B, Bennett JL, Marignier R, . Diagnosis of myelin oligodendrocyte glycoprotein antibody-associated disease: International MOGAD Panel proposed criteria. Lancet Neurol. 2023;22(3):268-282. doi:10.1016/S1474-4422(22)00431-8 36706773

[3] Ayroza Galvão Ribeiro Gomes AB, Kulsvehagen L, Lipps P, . Immunoglobulin A antibodies against myelin oligodendrocyte glycoprotein in a subgroup of patients with central nervous system demyelination. JAMA Neurol. 2023;80(9):989-995. doi:10.1001/jamaneurol.2023.2523 37548987 PMC10407763

[4] Thompson AJ, Banwell BL, Barkhof F, . Diagnosis of multiple sclerosis: 2017 revisions of the McDonald criteria. Lancet Neurol. 2018;17(2):162-173. doi:10.1016/S1474-4422(17)30470-2 29275977

