# Supplementary material for: Ongoing Challenges in the Diagnosis of Myelin Oligodendrocyte Glycoprotein Antibody–Associated Disease
Source: JAMA Neurol. 2023 Oct 10;80(12):1377–9. doi: 10.1001/jamaneurol.2023.3956 (PMC10565644; doi:10.1001/jamaneurol.2023.3956)
Supplement: Supplement 1. — eMethods [file jamaneurol-e233956-s001.pdf]

Supplementary Online Content

Lipps P, Ayroza Galvão Ribeiro Gomes AB, Kulsvehagen L, et al. Ongoing challenges in the diagnosis of myelin oligodendrocyte glycoprotein antibody–associated disease. *JAMA Neurol.* Published online October 10, 2023. doi:10.1001/jamaneurol.2023.3956

eMethods
eReferences

This supplementary material has been provided by the authors to give readers additional information about their work.

eMethods

Study participants

We analyzed data from a previously reported multicenter study including 1344 patients (1323 ≥ 18 years, 21 < 18 years) with confirmed or suspected central nervous system (CNS) demyelination (**Flowchart of study cohort and design**).[1] All patients included in challenge 1 were older than 18 years. One patient with clear-positive myelin oligodendrocyte glycoprotein (MOG)-IgG was excluded due to partly missing clinical information. Of the 134 patients included in challenge 2, 131 were older than 18 years and 3 were younger. Clinical, laboratory, and imaging data were collected as part of clinical routine. Missing data was reported in detail in a previous publication.[1] Analysis of available magnetic resonance images (MRI) was performed by a board-certified neuroradiologist (M.A.M.) blinded to the clinical and serological data. Medical data were reviewed and independently rated by two board-certified neurologists for their final diagnosis of myelin oligodendrocyte glycoprotein antibody-associated disease (MOGAD)[2] or multiple sclerosis (MS)[3] according to patients' demographic (age and gender), clinical (core clinical syndromes) and paraclinical (neuroradiologist reviewed MRI, MOG-IgG and cerebrospinal fluid [CSF]-specific oligoclonal bands [OCBs]) features.

Flowchart of study cohort and design

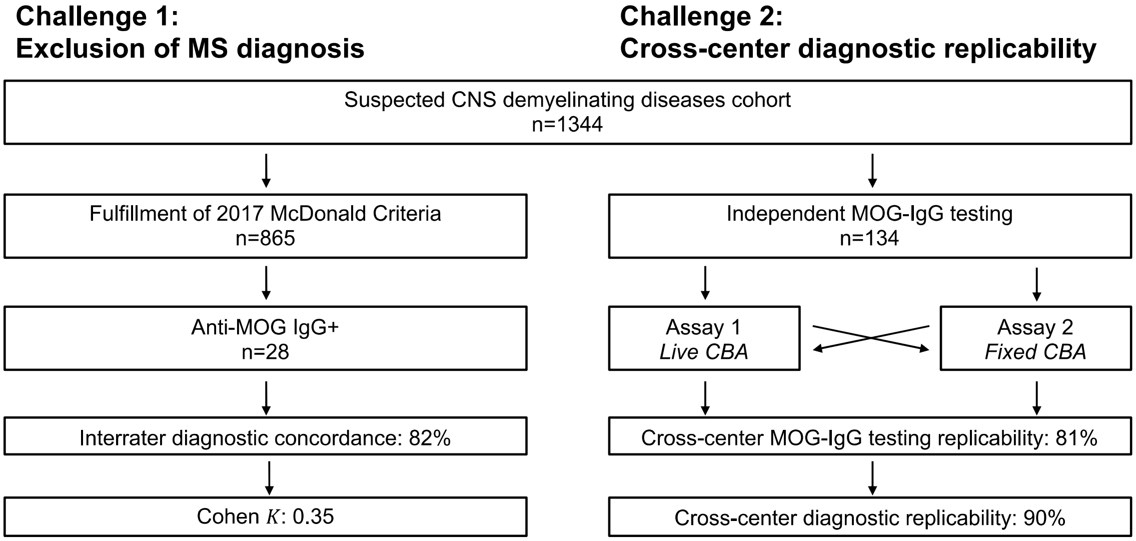

Measurement of myelin oligodendrocyte glycoprotein (MOG) immunoglobulin G (IgG) antibodies

<u>Assay 1 (live cell-based assay [CBA])</u>

Patients' sera (1:100) were tested for MOG-IgG using a live flow cytometry CBA with the human rhabdomyosarcoma cell line TE 671 stably transfected with the pRSV neo plasmid containing full-length human MOG (247 amino acids) or the empty vector as previously described.[1,4,5] Surface bound MOG-specific antibodies were detected with an IgG Fcγ fragment-specific secondary antibody (109-116-098, Jackson ImmunoResearch). For each serum sample, the geometric mean channel fluorescence intensity (geometric MFI) of the MOG cell line was divided by the geometric MFI of the control cell line to calculate a geometric MFI ratio, respectively. Data analysis was performed in FlowJo (10.6.2, Becton Dickinson and Company). The cutoff for low positive results was set to three standard deviations above the mean of a previously reported healthy cohort (geometric MFI ratio: ≥2.4 - <3).[1] Test results with

an additional surplus of 25% or above were considered clear positive (geometric MFI ratio ≥3).

<u>Assay 2 (fixed CBA)</u>

Patient sera were tested using a fixed cell-based assay according to the manufacturer's instructions (Euroimmun, Lübeck, Germany), as previously described.[6] In brief, sera were tested at increasing dilutions (1:10,1:100,1:1000) against full-length human MOG expressed on a HEK293 cell line fixed with formaldehyde and the respective control-transfected cells. Bound MOG-IgG was detected using a fluorescein-labeled anti-human IgG (Fc) secondary antibody and evaluated using fluorescence microscopy. The antibody titer was determined based on the fluorescence intensity at the above-mentioned dilutions as proposed by the manufacturer. Cutoff values were set to low positive (≥1:10 - <1:100) and clear positive (≥1:100).[2]

Data availability

Anonymized data are available on request from the corresponding author.

© 2023 Lipps P et al. *JAMA Neurology.*

eReferences

1. Ayroza Galvão Ribeiro Gomes AB, Kulsvehagen L, Lipps P, et al. Immunoglobulin A Antibodies Against Myelin Oligodendrocyte Glycoprotein in a Subgroup of Patients With Central Nervous System Demyelination. *JAMA Neurol*. Published online August 7, 2023. doi:10.1001/jamaneurol.2023.2523

2. Banwell B, Bennett JL, Marignier R, et al. Diagnosis of myelin oligodendrocyte glycoprotein antibody-associated disease: International MOGAD Panel proposed criteria. *Lancet Neurol*. 2023;0(0). doi:10.1016/S1474-4422(22)00431-8

3. Thompson AJ, Banwell BL, Barkhof F, et al. Diagnosis of multiple sclerosis: 2017 revisions of the McDonald criteria. *Lancet Neurol*. 2018;17(2):162-173. doi:10.1016/S1474-4422(17)30470-2

4. Pröbstel AK, Dornmair K, Bittner R, et al. Antibodies to MOG are transient in childhood acute disseminated encephalomyelitis. *Neurology*. 2011;77(6):580-588. doi:10.1212/WNL.0b013e318228c0b1

5. Pröbstel AK, Rudolf G, Dornmair K, et al. Anti-MOG antibodies are present in a subgroup of patients with a neuromyelitis optica phenotype. *J Neuroinflammation*. 2015;12:46. doi:10.1186/s12974-015-0256-1

6. Reindl M, Schanda K, Woodhall M, et al. International multicenter examination of MOG antibody assays. *Neurology - Neuroimmunology Neuroinflammation*. 2020;7(2). doi:10.1212/NXI.0000000000000674

© 2023 Lipps P et al. *JAMA Neurology.*
