# Supplementary material for: Ongoing Challenges in the Diagnosis of Myelin Oligodendrocyte Glycoprotein Antibody–Associated Disease
Source: JAMA Neurol. 2023 Oct 10;80(12):1377–9. doi: 10.1001/jamaneurol.2023.3956 (PMC10565644; doi:10.1001/jamaneurol.2023.3956)
Supplement: Supplement 2. — Data Sharing Statement [file jamaneurol-e233956-s002.pdf]

# Data Sharing Statement

Lipps. Ongoing Challenges in the Diagnosis of Myelin Oligodendrocyte Glycoprotein Antibody–Associated Disease. *JAMA Neurol*. Published October 10, 2023.
doi:10.1001/jamaneurol.2023.3956

**Data**
**Data available:** Yes
**Data types:** Deidentified participant data, Data (not involving human participants), Data dictionary
**How to access data:** Deidentified data will be shared upon request to the corresponding author: Anne-Katrin.Proebstel@usb.ch
**When available:** With publication

**Supporting Documents**
**Document types:** None

**Additional Information**
**Who can access the data:** researchers whose proposed use of the data has been approved
**Types of analyses:** for a specified purpose
**Mechanisms of data availability:** with a signed data access agreement
